# Evaluating the efficacy of a consumer‐centric method for ecological sampling: Using bonobo (*Pan paniscus*) feeding patterns as an instrument for tropical forest characterization

**DOI:** 10.1002/ece3.9606

**Published:** 2022-12-29

**Authors:** Erin G. Wessling, Liran Samuni, Roger Mundry, Miguel Adan Pascual, Stefano Lucchesi, Bienfait Kambale, Martin Surbeck

**Affiliations:** ^1^ Harvard University Cambridge Massachusetts USA; ^2^ St. Andrews, School of Psychology and Neuroscience University of St Andrews St Andrews UK; ^3^ Platform Bioinformatics and Biostatistics VetMedUni Vienna Austria; ^4^ Cognitive Ethology Laboratory, German Primate Center Leibniz Institute for Primate Research Göttingen Germany; ^5^ Department for Primate Cognition Georg‐August‐University Göttingen Göttingen Germany; ^6^ Leibniz Science Campus Primate Cognition Göttingen Germany; ^7^ Kokolopori Bonobo Research Project Tshuapa Democratic Republic of the Congo; ^8^ Max Planck Institute for Evolutionary Anthropology Leipzig Germany; ^9^ Centre de Surveillance de la Biodiversité de l'Université de Kisangani Kisangani Democratic Republic of the Congo

**Keywords:** dispersion, distribution, food availability, resource selection, species abundance, vegetation plot

## Abstract

Characteristics of food availability and distribution are key components of a species' ecology. Objective ecological surveying used in animal behavior research does not consider aspects of selection by the consumer and therefore may produce imprecise measures of availability. We propose a method to integrate ecological sampling of an animal's environment into existing behavioral data collection systems by using the consumer as the surveyor. Here, we evaluate the consumer‐centric method (CCM) of assessing resource availability for its ability to measure food resource abundance, distribution, and dispersion. This method catalogs feeding locations observed during behavioral observation and uses aggregated data to characterize these ecological metrics. We evaluated the CCM relative to traditional vegetation plot surveying using accumulated feeding locations across 3 years visited by a tropical frugivore, the bonobo (*Pan paniscus*), and compared it with data derived from over 200 vegetation plots across their 50 km^2^+ home ranges. We demonstrate that food species abundance estimates derived from the CCM are comparable to those derived from traditional vegetation plot sampling in less than 2 years of data collection, and agreement improved when accounting for aspects of consumer selectivity in objective vegetation plot sampling (e.g., tree size minima). Density correlated between CCM and plot‐derived estimates and was relatively insensitive to home range inclusion and other species characteristics, however, it was sensitive to sampling frequency. Agreement between the methods in relative distribution of resources performed better across species than expected by chance, although measures of dispersion correlated poorly. Once tested in other systems, the CCM may provide a robust measure of food availability for use in relative food availability indices and can be incorporated into existing observational data collection. The CCM has an advantage over traditional sampling methods as it incorporates sampling biases relevant to the consumer, thereby serving as a promising method for animal behavioral research.

## Graphical abstract

We propose a method to integrate ecological sampling of an animal's environment into existing behavioral data collection systems by using the consumer as the surveyor. Using a population of bonobos (*Pan paniscus*) as an exemplar, we demonstrate that food species abundance estimates derived from our method are comparable to those derived from traditional vegetation plot sampling in less than 2 years of data collection, and agreement improved when accounting for aspects of consumer selectivity in objective vegetation plot sampling (e.g., size minima). Once tested in other systems, our method may provide a robust measure of food availability for use in relative food availability indices, and has an advantage over traditional sampling methods as it incorporates sampling biases relevant to the consumer, thereby serving as a promising method for animal behavioral research.

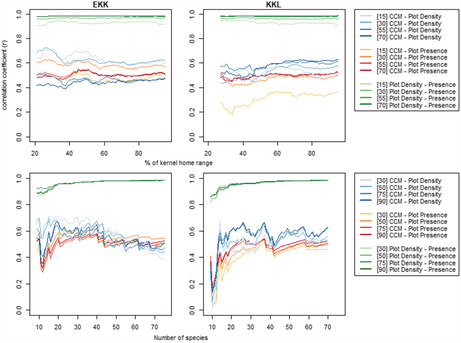

## 1 INTRODUCTION

The abundance, dispersion, and distribution of food resources not only determine species distribution but also have a strong impact on many aspects of an animal's life history, physiology, and sociality (Anholt & Werner, 1995; Chapman et al., 2015; Davies & Deviche, 2014; Hutto, 1990; Lambert & Rothman, 2015; Rogers, 1987; van Schaik et al., 1993; Vogel & Janson, 2007). Due to the core importance of food to an organism, the quantification of food availability and distribution are key considerations across studies and disciplines. Methods used to estimate food resource abundance, distribution, and dispersion are just as varied as the questions which necessitate these quantifications (Szigeti et al., 2016).

Measurement of the amount of food resources present for a consumer, such as *abundance or density* (i.e., estimation of the amount of a resource available in a landscape), depends heavily on the type of resource and scale of interest (Bowering et al., 2018; Morrison, 2016). Large‐scale analyses of abundance typically rely on remotely derived proxies via satellite imagery due to the practical impossibility to directly measure large areas of land. In such analyses, the abundance of resources available in a landscape is proxied from the estimation of land cover of preferred habitats (e.g., habitat‐based abundance) or includes methods like species distributional modeling (SDM) where the characteristics of locations occupied by a particular species are then used to extrapolate occupancy, and less frequently, abundance, over much larger scales.

For questions related more immediately to the consumer (at the individual or social group scale), direct measurement of exploitable resources offers more accurate insights into the resources available to a consumer (Foerster et al., 2016; Wessling et al., 2020). The abundance of mobile food resources may be measured via consumer behavior using metrics such as dietary composition, attack rates, feeding frequencies, and other metrics, under the assumption that consumption correlates with rates of resource encounter. Such metrics frequently serve only as proxies of food abundances in cases where measurements of true availability cannot be objectively or reliably inferred (Hutto, 1990; Lovette & Holmes, 1995; Watts & Mitani, 2015). However, for static food resources like plants, *abundance* is commonly estimated by cataloging the number of individual resources available to a consumer in subsets of an area of interest and extrapolating quantities to a global scale (e.g., plot, transect, or adaptive cluster sampling), or by sampling distances from predetermined or random points and extrapolating densities based on these distances (e.g., point‐centered distance sampling). Plot sampling is the most common sampling method in studies of frugivorous or folivorous animals, and involves the placement of randomly or systematically placed plots of fixed size across the area of interest, and all individuals contained within those plots are inventoried and frequently measured for size (e.g., trunk size; Baraloto et al., 2013; Ståhl et al., 2017; Vogel & Janson, 2007). A number of considerations feed into the selection of plot size, shape, placement, and number (Bonham, 2013), such as species form (e.g., tree, liana, and herb), dispersion (e.g., clumped or dispersed), and anticipated rarity, all of which necessitate different optimized designs.

Plot sampling also allows the calculation of other metrics commonly of interest to animal behaviorists, such as food distribution and dispersion. To estimate *distribution*, that is, a calculation of relative resource density across space within a landscape, plot sampling may be further stratified across a given area relevant to a consumer at various scales ranging from individuals to populations (e.g., home range, landscape, or region). Distribution provides information about where and how many individuals are located within a space rather than simply about the number of individuals available globally within that system. Measures of *dispersion* (i.e., patterns of clustering or patchiness), such as Morisita's index (Morisita, 1962), are used to quantify the clustering of resources over space within a landscape (Krebs, 1999; Stephens & Krebs, 1986). Resource clustering is often used in the contexts of understanding resource competition and socio‐ecological behavior (Vogel & Janson, 2011). Quantifications of food species dispersion are perhaps even more varied in practice and sensitive to the scale relevant to the consumer (Myers, 1978; Vogel & Janson, 2011). Dispersion metrics may also require distinct sampling methods tailored to specific questions (e.g., focal tree observation: Vogel & Janson, 2007, 2011), thus potentially require supplementary surveying efforts to plot sampling.

Despite its centrality to animal research, ecological sampling design to evaluate abundance, distribution, or dispersion frequently does not conform to recommended standards or is adequately validated by animal ecologists (Mortelliti et al., 2010; Szigeti et al., 2016). For example, while sampling effort can substantially impact measures of resource abundance, it is rarely validated whether sampling efforts are sufficient to adequately measure the intended metrics. Furthermore, ecological data collection often requires research effort in addition to ongoing behavioral observations and is time intensive and thus infrequently conducted. Snapshots of abundance (i.e., measurements of a landscape spanning short intervals of time) derived from these efforts may therefore be used up to decades after they have been measured without any consideration of changes that may have occurred since the last assessment. For example, primate research sites commonly approximate intra‐annual changes in food availability using species densities calculated from surveys that have not been updated since the establishment of ecological monitoring, in some cases representing significant time lags of 6 or more years (Klein et al., 2021; Potts et al., 2016; Wessling et al., 2018).

The problem of insufficient quantifications of resource availability also extends to sampling design. Traditional sampling methods in animal ecology, like plot sampling, are perceived as objective measures of the resources potentially accessible to a consumer. However, these methods are by design blind to aspects of resource selection by the consumer, and thus likely introduce an unknown measurement error. The distinction between resource accessibility and availability is important, as only the latter considers that not all individual food items are equally attractive to a consumer. As such, if researchers are interested in an accurate representation of the resources relevant to a consumer, then aspects of resource selection must be incorporated into resource availability estimates.

Ecological sampling is time intensive and the need to incorporate resource selection into resource availability metrics adds an additional burden on sampling methods. Given the inadequacies of existing methods for the estimation of an animal's food availability, is there a way to conduct ecological sampling that is time efficient within existing behavioral data collection systems and also integrates resource selection criteria of the consumer? Behavioral observation has been used extensively as a measure of food availability (Hutto, 1990; Lovette & Holmes, 1995), dispersion (Vogel & Janson, 2011), and preference (Forester et al., 2009; Kent & Sherry, 2020), however, these methods are either limited in application or still necessitate ecological data collection to be collected in parallel to behavioral observation. We therefore introduce a consumer‐centric method (CCM) for animal behavioral ecology studies which uses the consumer as the survey vehicle to quantify food resources in a landscape. With this method, researchers catalog discrete food resource locations (e.g., feeding tree locations) as they are consumed during the process of behavioral observation. The CCM compiles the geographic location of food resources visited by a consumer over a given time period to allow the calculation of ecological indices (e.g., proxies of density) similar to those collected in traditional ecological sampling (see below). The method uses the consumer as a surveyor that, over time, aggregates the locations of all species in their diet within a given area of interest to that consumer (e.g., home range).

Data reliant upon patterns of usage by a consumer, such as that proposed in the CCM, would result in a form of a presence‐only dataset that mimics the logic employed in species distributional modeling (SDMs). In other words, aggregations of data on species' presence can be used to generate broader‐scale predictions of relative abundance (i.e., distribution; Gomes et al., 2018). The proliferation of presence‐based datasets for uses like SDMs is indicative that there is significant utility in these types of data for estimating species distribution, and presence‐only datasets have been further employed to estimate species abundances with mixed success (Bradley, 2016; Gomes et al., 2018; Gutiérrez et al., 2013; Hwang & He, 2011; Jiménez‐Valverde et al., 2021; Royle & Nichols, 2003; Yackulic et al., 2013). The CCM operates similarly to these applications, in that behavioral observation would contribute data on feeding locations that subsequently translate into presence‐based data on dietary species over a smaller spatial scale.

The CCM combines aspects of presence‐based abundance modeling outlined above with those of animal consumption rate proxies (e.g., prey attack rates), and consequently cannot be used universally in all animal consumer applications. First, as the CCM relies on the aggregation of data over time, data collected on resource locations must be representative of the species' same distribution over time, and therefore the method must only be applied to estimate resources that are immobile, discrete, and spatially explicit entities. Second, to allow for data accumulation, the CCM can only be applied to consumers who reuse the same space over time, such as a home range, and assumes that they have equal access to all the areas of this space (Alldredge et al., 1998). The data afforded by the CCM represent the outcome of both consumer‐independent food abundance as well as consumer resource selection, therefore we must also assume that potential resource presence and consumer preference do not change within the period until adequate sampling has occurred (Manly et al., 2007). Subsequently, the CCM will likewise not be applicable to cases where objective measurements of resource abundance are necessary (as is the case with questions pertaining to, e.g., resource preference).

Consequently, because the CCM reflects the outcome of both selection and environmental conditions, it conflates the two, and therefore its estimates cannot be detached from either input (Kent & Sherry, 2020). Similar to the use of consumption rate proxies, this format, therefore, is also limited in the types of questions such a methodology can be applied to, specifically in cases where questions of selection are important. However, the CCM differs from consumption rate methodologies, in that like presence‐only models, it is location based and does not conflate encounter rate with metrics of abundance. With an understanding of the constraints of the method, there is precedent to extrapolating presence‐based datasets to infer global patterns of abundances and distribution. Therefore, we ask, in cases where researchers do not need to understand patterns of selection by a consumer but simply its outcome (e.g., fruit availability indices), can the CCM replace plot‐based data collection?

To answer this question, we evaluate the CCM relative to traditional plot‐based ecological data collection using accumulated feeding locations from two social groups of a tropical frugivore, the bonobo (*Pan paniscus*), as a case study. Unlike existing metrics of food abundance derived from behavioral observation (e.g., prey attack rates), which can only serve as proxies of difficult‐to‐measure food abundance, in the case of the CCM, we can evaluate the estimates provided by the CCM against availability estimates provided by more traditional plot sampling. Specifically, we investigated whether behavioral data on feeding locations (trees and lianas) provide a reliable dataset allowing inference about food species' (1) densities, (2) distribution, and (3) dispersion. We additionally assess (4) the minimum sampling effort required and (5) for what characteristics of a food species this method can be considered most reliable.

## 2 METHODS

### 2.1 Study species and behavioral observation

**FIGURE 1:**
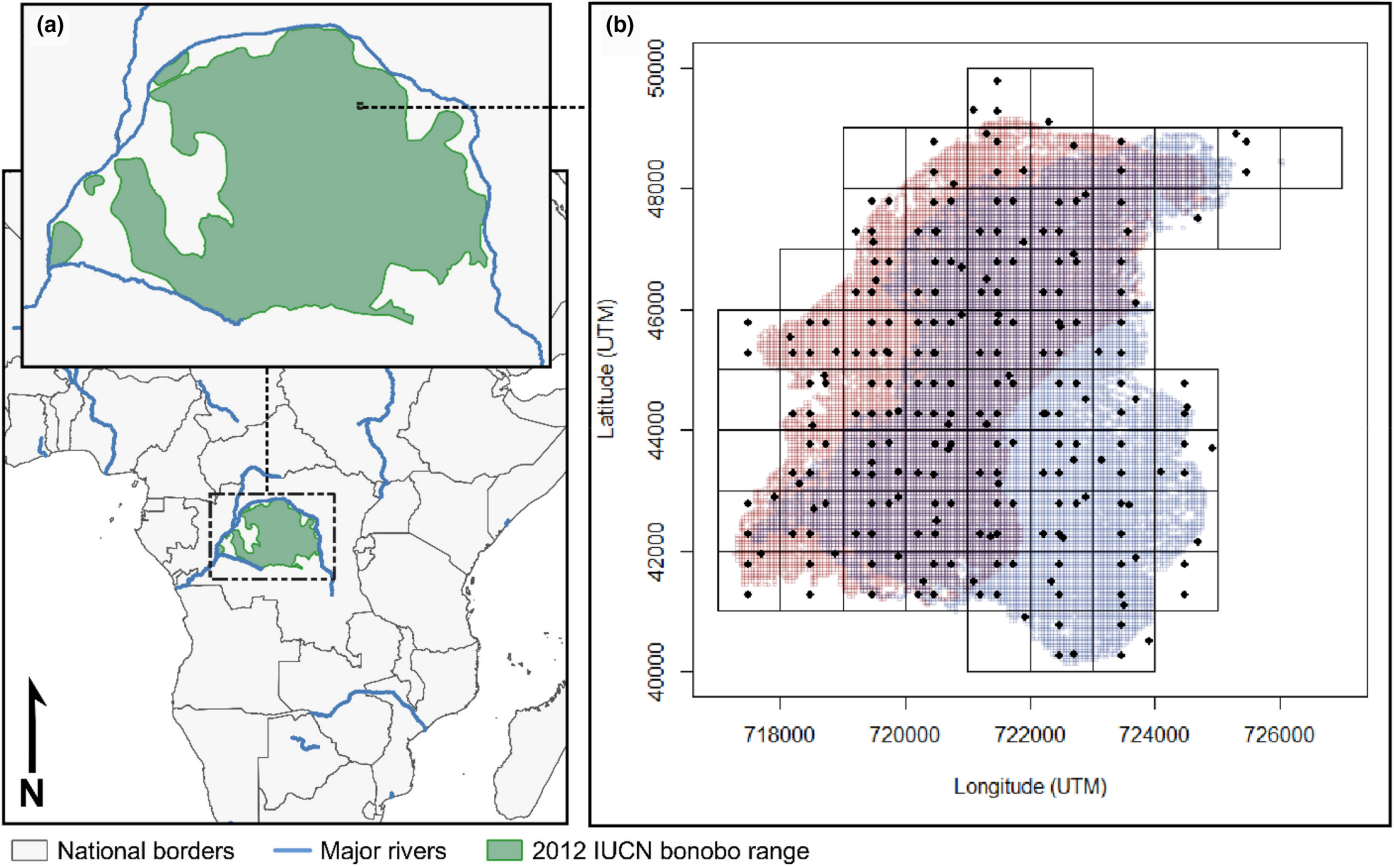
(a) Location of the study site relative to global bonobo distribution. (b) 50 × 50 m habitat plots (black dots; not to scale) within 1 km^2^ grid cells (black square) overlaid upon all visited 50 × 50 cells within the 95% home range kernels for Ekalakala (red squares; to scale) and Kokoalongo (blue squares; to scale) bonobo groups.

Data were collected at the Kokolopori Bonobo Reserve (Figure 1) on two social groups of bonobos (Ekalakala: EKK, Kokoalongo: KKL) between May 2016 and December 2019. Like much of the bonobo range, the Kokolopori Bonobo Reserve is a mixture of monodominant and heterogenous continuous forest, interspersed with areas of permanent and seasonally inundated swamp (Surbeck et al., 2017). It is floristically rich, however, relatively skewed toward a handful of dominant arboreal species, most of which are consumed by bonobos (Section 2.4 in Supporting Information). Bonobos are predominantly frugivorous, focusing the majority of their diet on ripe fruits from trees and lianas within their home range, although they regularly consume a variety of other plant and animal food items including flowers, leaves, insects, honey, small mammals, underground truffles, and terrestrial herbaceous vegetation (Hohmann & Fruth, 2007; Lucchesi, Cheng, Wessling, et al., 2021; Sakamaki et al., 2016; Samuni et al., 2020). Bonobos live in a fission–fusion social system in which group members divide and range into subgroups of varying sizes and compositions independently throughout the day (Kuroda, 1979; Samuni et al., 2022). Average subgroup sizes accounted for approximately 63% and 28% of each social group (Section 2.1 in Supporting Information). Subgroups were followed daily for behavioral data collection over the course of a full day of activity (Section 2.1 in Supporting Information), during which we collected data on each tree or liana fed upon by a member of the observed bonobo group, including location of the trunk of the resource using a GPS (Garmin GPSMAP 62) and diameter at breast height for all feeding trees ≥20 cm and lianas ≥5 cm diameter in size (Sections 2.1 and 2.2 in Supporting Information). We ignored all feeding patches smaller than this minimum, as this was also the minimum diameter used in vegetation plot surveying. All independent individuals of both groups were present during a significant proportion of the data collection for this study, therefore the feeding behavior summarized in our dataset is also representative of all members of both social groups.

Due to GPS measurement error (commonly 15–20 m) and consequently an inability to distinguish individual trees on a small scale, we summarized feeding tree locations of each group into the presence or absence of each species in 50 × 50 m “observational cells” (Figure S1), in order to later relate these cells (in which species were present) to cells that had been visited by the bonobo group within the dataset but no individual of that species was visited. 50 m × 50 m cell size was chosen because it accommodates average GPS error between two points (potentially 40 m if GPS error is 20 m) and is comparable to the size of our vegetation plots. In practice, this resulted in a presence–absence database of observational cells for each species in the bonobo home ranges. We used location data collected with the GPS tracklog function to calculate the home range of both bonobo groups using kernel density estimates (see Section 2.1 in Supporting Information). These groups share overlapping areas of their home ranges, including 63% of the home ranges of both groups. We evaluated whether feeding location datasets were sufficiently sampled and stable by considering accumulation patterns of data per species over time (Section 2.2 in Supporting Information).

### 2.2 Vegetation plots

We conducted vegetation plot sampling by overlaying 1 × 1 km grid cells over the whole ranging area to identify relevant sampling scope and aimed to conduct plot sampling in every grid cell utilized by at least one of the groups (Figure 1 and Figure S1; Section 1.2 in Supporting Information). We sampled from within the overlaid 1 km^2^ cells to distribute sampling plots evenly throughout the home ranges of the groups, and to permit the compilation of different sampling schemes between data collectors (see Supporting Information for details). Like the observational cells, all vegetation plots were 50 × 50 m in size, within which data were collected on all trees meeting the minima defined for individuals in the observational cells. In total, we sampled 236 plots within these 1 km^2^ grid cells, of which 214 plots fell within the 95% home range of either group, with 162 and 170 within the 95% range of EKK and KKL, respectively (Figure 1). Plot sampling averaged 4.1 ± 1.6 (SD) plots per km^2^ (range: 1–7) and was determined to be of sufficient sampling depth (Section 1.3 in Supporting Information).

### 2.3 Comparison of datasets

#### 2.3.1 Density

To compare estimated species densities derived from each dataset (CCM or vegetation plots), we derived three different indices (Section 3.1 Equations 1–3 in Supporting Information). (1) We used the bonobo observational data to create a “presence index” based on bonobo feeding locations for each food species, estimated as the number of 50 × 50 m cells in which each species was present, divided by the total number of cells within the 95% kernel home range of each group (see Figure S6 for an example; hereafter “CCM Index”). (2) We calculated species density estimations using the vegetation plot data as the total number of individuals observed per area surveyed (num. individuals/km^2^, hereafter “Plot Density”). (3) We calculated the number of 50 × 50 m vegetation plots in which each species was present per total number of vegetation plots sampled for a more direct comparison with the CCM (hereafter “Plot Presence”).

To evaluate method agreement, we created pair‐wise sets of comparisons of the three density indices by means of Pearson's correlation tests and used the correlation coefficient (*r*) as a measure of the strength of agreement between methods. We conducted the pair‐wise comparisons while assessing the influence of sampling effort on method agreement by varying levels of home range usage (kernel % range from 20% to 95% in increments of 1%) and dietary inclusion (top 10 most consumed species until full diet) for each group. We only considered comparisons with at least 10 species in at least 10 vegetation plots as a means of imposing minimum thresholds necessary to avoid distortion of comparison metrics due to small sample sizes. We chose 10 species as a minimum threshold as this is a commonly recommended minimum sample size for basic regression analyses (Gotelli & Ellison, 2004). To evaluate potential biases in spatial coverage of sampling by the two methods, we additionally created a moving window over the kernel home range from 20% to 95% for which to compare methods more directly according to home range location. This window accounts for variation in area coverage by adjusting the window radius to impose similarly sized datasets for comparison over the range of % kernel inclusion (i.e., for agreement from home range core to the periphery; Section 3.1 in Supporting Information).

Finally, to identify potential dataset minima required for reliable and stable density indices derived from the CCM (i.e., temporal duration of dataset aggregation), we evaluated the pattern of correlation strength between indices from each method as the dataset grew over time (i.e., day of data collection), and set the minimum as the point from which the correlation coefficient remains relatively stable. We describe *p*‐values for these correlations in our summaries below; however, as these correlations require independent data and because we evaluated thousands of correlation coefficients per group (*n*_EKK_ = 15,075 and *n*_KKL_ = 12,834), we do not draw inference based on *p*‐values but instead focus only on correlation coefficients.

#### 2.3.2 Dispersion

To evaluate agreement between methods in characterizing food species dispersion, we used Morisita's index (Morisita, 1962). Morisita's index is a statistical index that measures dispersion (i.e., clustering) across a sample set, providing a measure of the likelihood of samples within that sample set to be of similar composition. To allow for standardized and directly comparable sample units from which to calculate this index for both methods, we aggregated the number of individuals per species visited by the bonobos across three different grid cell sizes (500 × 500 m cells, 1000 × 1000 m cells, and 1500 × 1500 m cells), and calculated the average number of individuals for each species in each of these grid cells using the vegetation plot dataset. We chose this range of cell sizes because they provide a compromise between allowing for at least one vegetation plot to be sampled within each cell and considering the number of cells to be available for comparison between methodologies, while remaining reasonably biologically relevant to our study species (e.g., 8 km travel distance per day [Lucchesi, Cheng, Deschner, et al., 2021] and 30 km^2^+ home range [this study, Samuni et al., 2020]). For both datasets, we then calculated Morisita's index using the *dispindmorisita* function of the package ‘vegan’ (Oksanen et al., 2019) for each species. We further accounted for an unusual distribution of Morisitia's indices deriving from the vegetation plot dataset that exaggerated the scale of the range of data and therefore obscured meaningful comparison between datasets, by ad hoc transforming the data to allow for a more normal distribution (Section 3.2 in Supporting Information).

#### 2.3.3 Distribution

To evaluate the efficacy of the CCM to reliably quantify the distribution of food species in a landscape, we aggregated data by grid cell as was similarly done in our dispersion comparison. We compiled the data for both the observational cell and vegetation plot datasets in two ways: by either (i) aggregating (CCM) or averaging (plot dataset) the number of individuals per species per grid cell or (ii) by marking the presence/absence of a given species per grid cell size. We chose to average rather than aggregate plot data because greater plot sampling in a grid cell will inherently increase estimates of species densities, whereas sampling biases in CCM could be accounted for by controlling for location within each group's home range (i.e., % kernel home range). We then fitted model sets separately for each cell size and group (six sets of up to 70 species each), using each food species as a dataset and each cell as a datapoint. We used the estimated bonobo feeding data abundance per cell (a measure of distribution) as the response and the plot abundance as the test predictor using zero‐inflated Poisson models (500 × 500 m grid size) or simple linear models for 1000 × 1000 and 1500 × 1500 m grid sizes. Within these models, to account for variation in home range utilization by the bonobos, we controlled for the % kernel home range of each cell by averaging the % kernel value assigned to each of the vegetation plots used to estimate the species abundance within that cell. We then calculated average Nagelkerke's *R* ^2^ (500 × 500 m) or *r* ^2^ (1000 × 1000 m and 1500 × 1500 m) for each model set across levels of dietary inclusion (see Section 3.3 in Supporting Information for detailed descriptions of the fitted models and model checks).

To also evaluate agreement between methods on simple presence of a species in a cell, we fitted a generalized linear mixed model with binomial error structure (Baayen, 2008) for each grid cell size and each social group. The response in this model was the presence or absence of a species in a given cell as predicted by the bonobo observational data (with one datapoint per species per cell), and presence as measured by vegetation plot and % kernel as test predictors. In these (six total) binomial models, we included cell ID and species as random effects and included random slopes for presence/absence in the plots and their correlation within the random effect of species (Section 3.3 in Supporting Information for details and model checks). As a last validation of distribution agreement, we identified when bonobos missed the presence of a species in a cell that had been identified in the vegetation plots and calculated a proportion of missed species occurrences out of all cells per species, as well as evaluated potential sources of biases in likelihood to miss a species in a cell (see Section 2.4).

### 2.4 Identifying sources of bias

If a consumer is selective in which resources it uses within a landscape, then measurements from vegetation plots may not accurately measure the relevant resources to that consumer. To evaluate these potential discrepancies, we compared food tree and liana sizes (strongly tied to variability in food crop production: Chapman et al., 1992; Section 4 in Supporting Information) between CCM and vegetation plot data as an example of a potential selective characteristic. We then quantified seven characteristics of each species to evaluate how they contribute to rates of data accumulation and agreement between our sampling methods. Specifically, we considered the lifeform (tree or liana), patterns of dispersion, consumed food item (fruit or non‐fruit), seasonality of consumption, density in the landscape, DBH variability, and frequency of consumption (Section 4.1 in Supporting Information) as test predictors in models with the following responses (Section 4.2 in Supporting Information): (1) the speed at which data accumulate in the CCM dataset, (2) a measure of the difference between estimates of density between the methods, and (3) likelihood for bonobos to miss the presence of a species in a cell (Section 4.2 in Supporting Information).

### 2.5 General analyses

All data analyses were conducted in R (version 4.0.2; R Core Team, 2020), and models were fitted using functions of the ‘lme4’ package (1.1.23; Bates et al., 2015). We report *p*‐values between .05 and .1 as a “trend” for all models to ease issues of dichotomization of significance (Stoehr, 1999). To avoid issues of multiple testing when identical models were run across responses that varied only in their summary method (e.g., grid cell size) or dataset (e.g., social group), we describe only patterns that are stable and significant or trending across at least half of each model set; full results for all models as well as further description of all methods and model checks can be found in the Supporting Information. We log‐transformed predictor (e.g., species density and consumption frequency) and response (all density indices) variables with significant skew to prevent issues with model fit (e.g., overdispersion, residual distribution, and leverage).

## 3 RESULTS

### 3.1 Consumer‐centric dataset

The bonobo groups visited (i.e., fed in) a total of 12,430 (EKK) and 13,827 (KKL) 50 × 50 m cells, amounting to an area “surveyed” of 31.1 (EKK) and 34.6 km^2^ (KKL). This amounts to 58.6 km^2^ total area surveyed, as 46.7% of this area occurred within the home range overlap of both communities. Bonobos from EKK and KKL fed on a total of 78 tree and liana species (88.6% occurring in the diets of both groups) from trees and lianas, of which 96% of feeding occasions could be identified to a local name. These observations amounted to 8818 (EKK) and 9140 (KKL) unique feeding tree/liana locations (50 × 50 m) consisting of 76 (EKK) and 72 (KKL) species, of which 58 (EKK) and 55 (KKL) species were consumed in at least 10 locations. The diets of both groups were strongly skewed toward a few frequently consumed species (Section 2.3 in Supporting Information). The groups visited a similar number of locations each day, with a mean of 10.0 ± 5.5 (KKL) and 8.9 ± 5.0 (EKK) locations visited. On average, 4.5 ± 2.0 (KKL) and 4.3 ± 1.8 (EKK) species were consumed per day by the bonobos.

Bonobos visited 60% (EKK) and 56% (KKL) of all visited cells within the first year of data collection, with gradual declines in the accumulation of newly visited cells over the 3+ years study period in both groups and a clear approach toward an asymptote for most of the top 30 species (Figure S3). We found that the speed at which new feeding locations were added to the dataset also decreased across species (i.e., longer accumulation times) with each passing year for both groups, and that much of the observed decrease in new locations visited over time was likely driven by significant gains early within the dataset (Figures S3 and S4; Section 2.2 in Supporting Information). Data on species more variable in size (DBH) accumulated slower in EKK than species more uniform in size (but no such relationship was found in KKL), and accumulation was also slower in species consumed for their fruits and in more abundant species in the landscape in both groups (Section 4.3 in Supporting Information, Tables S2 and S3).

### 3.2 Vegetation plot dataset

In total, 14,855 trees and lianas were measured across 214 habitat plots (Section 1.1 in Supporting Information), thus exceeding plot surveying minima (124 plots for this dataset, Section 1.3 in Supporting Information). Plot surveying required a cumulative total of 146 team days, averaging 1.7 ± 0.6 (SD) plots completed per team day (range: 1–4). Trees comprised the majority (66.9%) of the individuals measured. This dataset averaged 277.7 individual trees and lianas/ha across the habitat of these two groups, with 196.1 indiv./ha for food species and 168.2 indiv./ha for potential food trees that met bonobo size minima (see below) for the EKK and KKL home ranges collectively.

Seventy‐five of the 200 taxa identified in the plots were consumed by at least one of the two groups, with 67 of 72 (EKK) and 70 (KKL) of 75 species in the Kokolopori bonobo diet occurring in the plots. Like the bonobo diet, the forest was heavily biased toward a few species, with one species accounting for over 10% of the dataset (“Bofili”, local name for *Scorodophloeus zenkeri*), and the top 10 most common tree species accounting for almost 40% of all trees and lianas (*n* = 6375, 39.2%). Correspondingly, only 16 species account for over 50% of the individuals in the plots, of which 11 occur in the diet of both groups. Species in the bonobo diet accounted for 67% of the total number of trees or lianas observed in the Kokolopori landscape.

### 3.3 Dataset comparison

#### 3.3.1 Consumer selectivity of tree sizes

Trees visited by bonobos were significantly larger in diameter on average than trees measured in the plots (EKK: *t* = −17.71, *p* < .001; KKL: *t* = −20.38, *p* < .001), but by only an average of <1 cm in both groups (Table S1). For 23.1% of consumed species, we found more individuals in the plots that did not reach the minimum size consumed than those who did exceed this minimum threshold. We subsequently restricted all analyses to trees/lianas that met bonobo size thresholds, consequently reducing the number of individuals included in the plot dataset by approximately 18% for the home ranges of both groups (8891 individuals in EKK and 8685 in KKL; Section 4 in Supporting Information). Reducing the dataset had a measurable effect on the correlation strengths between estimates of density (see below), with an average improvement of .04 for comparison (*r*) of the CCM estimate with the Plot Presence estimates and .07 improvement in correlation coefficient in the comparison of the CCM estimate with Plot Density.

#### 3.3.2 Density

**FIGURE 2:**
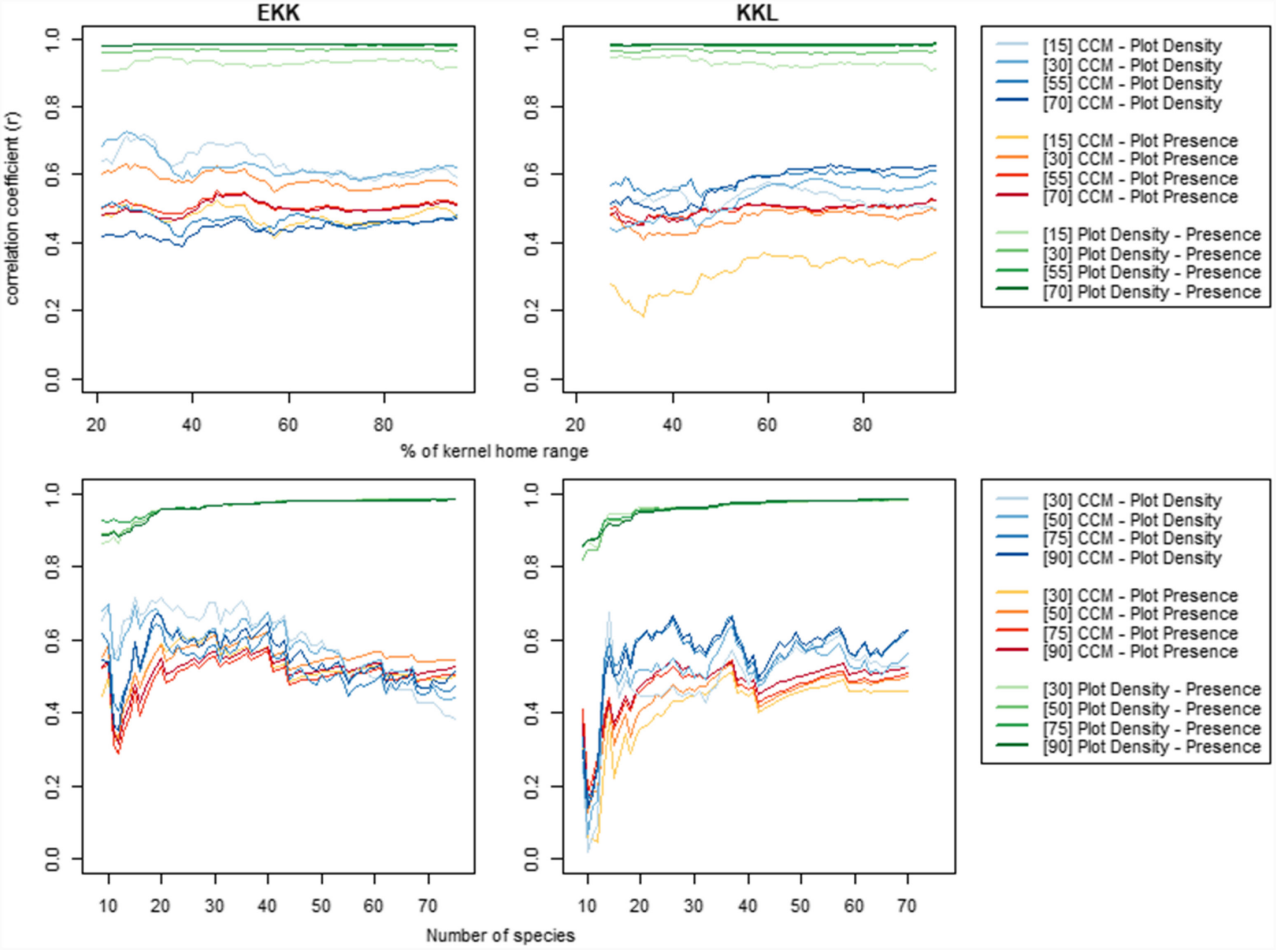
Correlation coefficients of density estimates between sampling methods (i.e., CCM and vegetation plots) for EKK (left) and KKL (right), according to home range percentage (top) and dietary inclusion (bottom). Color groups depict the three comparisons in this study (see legend), with numbers in brackets indicating number of species included (top legend) or percent home range included (bottom legend).

We found that the density estimates from the CCM and vegetation plots were comparable in both groups (Table 1 and Figure 2). Patterns of correlational strength between the methods stabilized and smoothed from approximately 50% kernel home range inclusion and above, and when approximately a minimum of 15 species was included in the dataset of both groups. Statistical significance of the correlation was reached in both groups when including ca. 20 of the top species or more. The inclusion of less frequently used areas of the home range in the comparison did not appear to considerably affect the strength of agreement between methods but correlation strength decreased with greater number of species included in the comparison (Figure 2; Table 1). While we did observe that peripheral areas of the home range generally resulted in lower methodological agreement (Figure S6), bonobo data appeared largely insensitive to inclusion of the outer reaches of the home range in both groups when included alongside more intensively surveyed areas (i.e., the core range).

**TABLE 1:** Summary of correlation coefficients (*r*) between density estimates derived from the CCM and vegetation plot sampling for all significant comparisons above 50% kernel home range and of at least 10 species

	*r*_mean_ ± SD (all combinations)	*r*_range_ (all combinations)	Number of species with highest r_mean_ (r_mean_)^a^	Number of species at *r*_max_	% kernel with highest *r*_mean_ ^b^	% kernel at *r*_max_
(a) Kokoalongo						
Plot Presence and Plot Density	0.96 ± 0.02	0.82–0.99	–	–	–	–
Plot Presence and CCM	0.48 ± 0.04	0.31–0.55	36 species (0.53)	10	94% (0.50)	58
Plot Density and CCM	0.58 ± 0.04	0.46–0.69	36 species (0.65)	71	69% (0.60)	51
(b) Ekalakala						
Plot Presence and Plot Density	0.97 ± 0.06	0.88–0.99	–	–	–	–
Plot Presence and CCM	0.52 ± 0.02	0.31–0.63	40 species (0.58)	48	51% (0.56)	95
Plot Density and CCM	0.54 ± 0.03	0.42–0.70	19 species (0.66)	26	50% (0.58)	95

^a^ Averaged across all combinations of % home range inclusion per number of species included.

^b^ Averaged across all combinations of number of species included per % home range inclusion.

Broadly, the CCM more closely matched estimates of Plot Density relative to Plot Presence. However, for both comparisons, we observed a decrease in the correlation coefficient the greater the number of species included in the EKK dataset (Figure 2, blue lines in bottom left panel). For both groups, we found highest agreement between methods when restricting the comparison to the top 36–40 species (i.e., approximately half of the species in the diet), with one exception that only slightly outcompeted the r of the same range (KKL CCM vs. Plot Density). As expected, comparison between Plot Density and Plot Presence remained consistently high regardless of location within the home range of the bonobo groups, although correlations were lower when fewer species were included.

Once our moving window reached the dataset minimum of 20 plots at ca. 30% kernel, the correlation coefficient of the CCM with plot estimates increased until they reached a maximum of around 60% kernel home range in both groups (Figure S6). Peripheral areas of the home range were generally lower in agreement than more central areas but did not show persistent decreases with increasing peripheralization in a manner that would suggest consistently poorer sampling in peripheral areas. Sampling agreement was strongest within our moving windows for the most frequently consumed species (e.g., 15 or 30 species) relative to more comprehensive subsets of the two groups' diets (e.g., 55 and 70 species).

The density of the species in the landscape and the variability in size significantly impacted agreement between the methods (Table S4); specifically, lower species density in the plots (estimate average: 0.57 ± 0.11 [SE]) and lower size variability (−1.29 ± 0.62 [SE]) improved method agreement. Further, in KKL only, greater seasonality, non‐fruit item consumption, and greater consumption frequency decreased agreement between methods.

**FIGURE 3:**
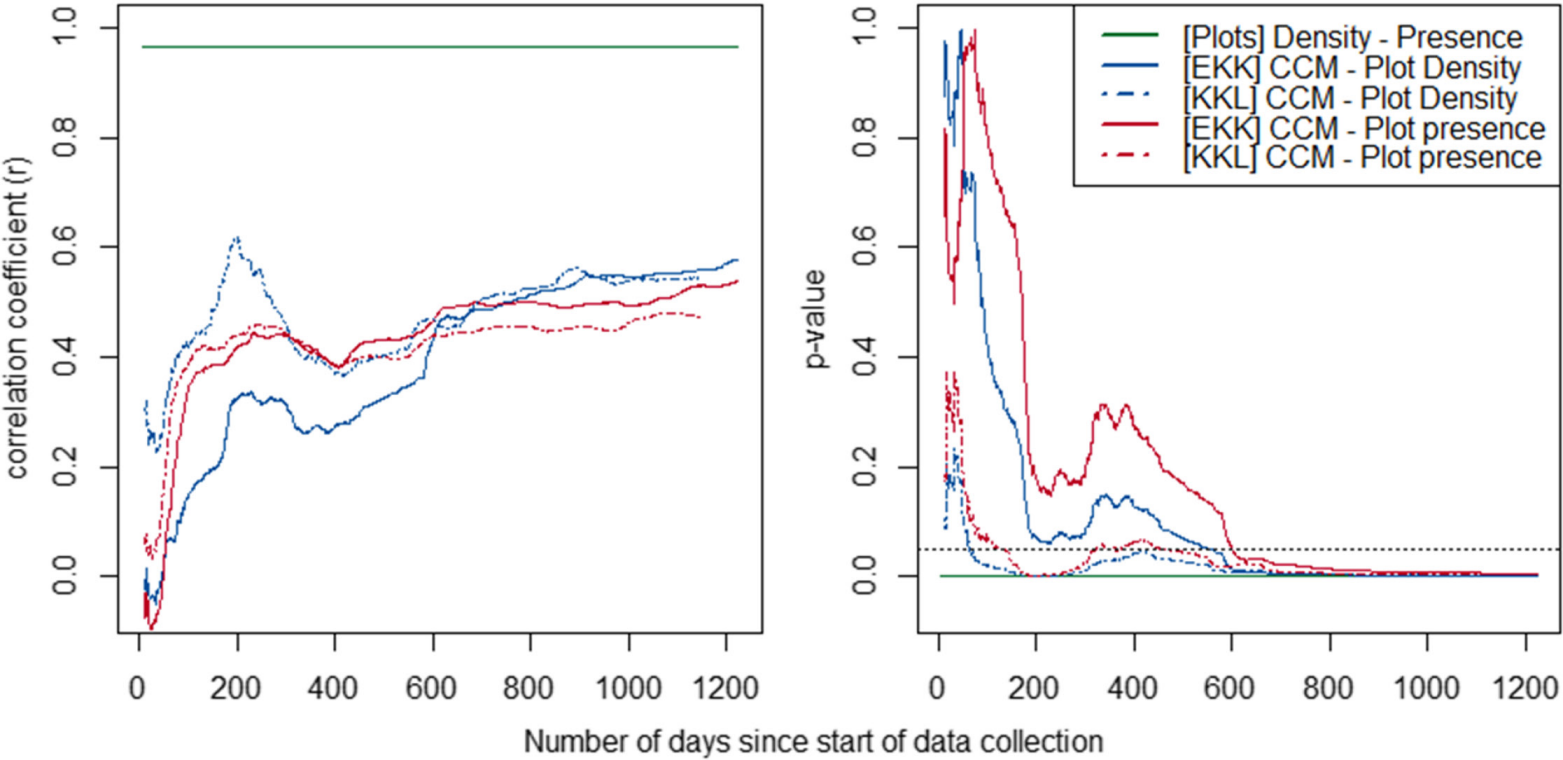
Pearson's *r* (left) and *p*‐value (right; dashed line indicates .05 alpha level) of all three methods comparisons (see legend) for Ekalakala (full line) and Kokoalongo (dashed lines) over the duration of the dataset.

Correlation strength between the two methods reached significance and stabilized across methods and groups once exceeding 600 days (i.e., ca. 5300 [KKL] to 6000 [EKK] total visited locations) and continued to improve as data were collected until the end of our data period (Figure 3; EKK_max_: 1222 days, KKL_max_: 1151 days). Similar correlational strengths were achieved briefly around the 200th day of data collection, however, its instability as data continued to aggregate suggests this brief peak in performance may have been an artifact of sampling rather than a reliable sampling minimum.

#### 3.3.3 Dispersion

Overall, Morisita's indices from the CCM correlated weakly and non‐significantly to vegetation plot indices, regardless of grid cell size used or bonobo group (Table 2a).

**TABLE 2:** Average (a) correlation coefficients (*r*) and (b) proportion of variance explained (*r*; 500 × 500 m) or Nagelkerke's *R* (1000 × 1000 m and 1500 × 1500 m) between the CCM and plot datasets across three different grid cell sizes for (a) dispersion and (b) distribution estimates

(a) Dispersion					
	Ekalakala		Kokoalongo		
Cell size	Mean + SD (range)		Mean + SD (range)		
500	0.08 + 0.17 (−0.54, 0.55)		0.00 + 0.16 (−0.35, 0.61)		
1000	0.00 + 0.19 (−0.8, 0.25)		−0.03 + 0.14 (−0.65, 0.13)		
1500	−0.17 + 0.14 (−0.86, 0.07)		−0.20 + 0.14 (−0.83, −0.01)		
(b) Distribution					
	Ekalakala		Kokoalongo		
Cell size	Mean ± SD (range)	Num species *p* < .05 (% of total species)	Mean ± SD (range)	Num species *p* < .05 (% of total species)	Significant species in both groups
500	0.25 ± 0.05 (0.21, 0.38)	15 (29%)	0.23 ± 0.04 (0.20, 0.36)	13 (28%)	11
1000	0.23 ± 0.02 (0.20, 0.30)	13 (19%)	0.24 ± 0.02 (0.20, 0.31)	8 (11%)	7
1500	0.23 ± 0.03 (0.14, 0.27)	8 (12%)	0.24 ± 0.02 (0.21, 0.27)	6 (9%)	3

#### 3.3.4 Distribution

**FIGURE 4:**
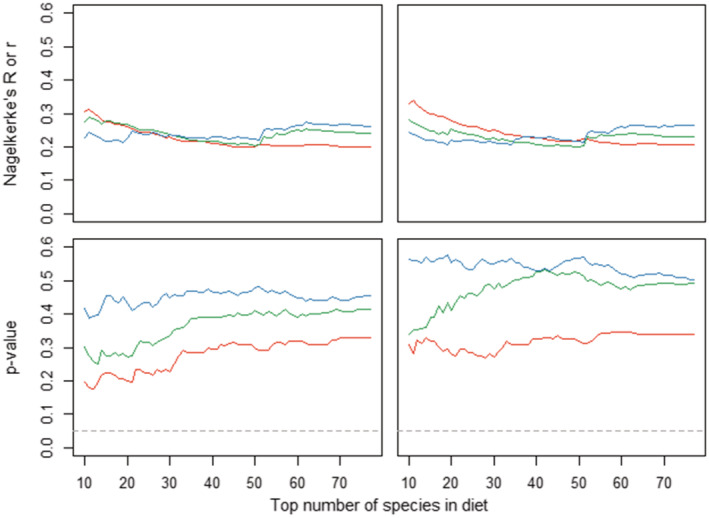
Averaged proportion variance explained *r* or Nagelkerne's *R* (top) and *p*‐values for the estimate (bottom; dashed line indicates .05 alpha level) for correlations between estimated abundances per cell of species (i.e., distribution agreement) as derived from CCM and vegetation plots for EKK (left) and KKL (right) and for three grid cell sizes (red: 500 × 500 m, green: 1000 × 1000 m, and blue: 1500 × 1500 m).

Across both bonobo groups and all three grid cell sizes, we found that more species significantly correlated between the two methods for individual abundances across cells than would be expected by chance, with an average of 18% of species significantly correlated between methods across the three cell sizes (Table 2b). The percentage of species with significant correlations across methods declined as grid cell sizes increased, as did the number of significant species which remained consistent across both groups. Generally, proportion variance explained (*r* or Nagelkerke's *R*) by abundance per cell based on plots averaged 0.25 ± 0.32 [SD] across species in all grid cell sizes and groups for predicting abundance per cell based on CCM. Average *r* did not vary substantially with cell size or between groups (Figure 4).

The presence of a species in a cell as measured by plots significantly predicted the presence of that species in the cell as identified with CCM (estimate: 0.60 ± 0.20 (SD), range: 0.32–0.81; Table S5). The location of a cell within the home range appeared to play a consistent role, with food species less likely to be identified by CCM in more peripheral cells (average estimate: −0.05 ± 0.01 (SD), range: −0.05 to −0.04; Table S5). Bonobos missed the presence of a species on average in 17.5% ± 16.3% (SD; range: 0.0%–68.4%) of the 500 × 500 m cells and in 18.4% ± 16.5% (SD; range: 0%–61.2%) of the 1000 × 1000 m cells. Increases in overall species densities correlated with an increase in the likelihood for bonobos to miss the presence of species in a cell irrespective of cell size or group but species were less likely to be missed in a cell if they were more frequently consumed. We additionally found some support for species consumed for their fruits to be more likely to be missed in smaller cell sizes (Table S6).

## 4 DISCUSSION

Here, we demonstrate the applicability of the consumer‐centric method (CCM) for measuring resource density and distribution in an animal's landscape. We demonstrate that food species availability estimates derived from the CCM method are modestly comparable to estimates derived from traditional vegetation plot sampling following a relatively short data collection timeframe, including before data have reached saturation. The method also shows promise for characterizing the distribution of food patches within a landscape, but current analytical power was likely insufficient to adequately evaluate this relationship. Furthermore, we argue that the CCM has an advantage over traditional sampling methods for some research questions as it incorporates sampling bias important to the consumer into the quantification of the ecological landscape. We discuss the consequences of this advantage regarding the utility of the CCM in studies of animal ecology.

### 4.1 Robustness of the CCM

The CCM estimates of density showed moderate similarity to estimates from traditional ecological sampling. Behavioral ecologists have previously used consumption rates to infer about the abundance of food resources (Hutto, 1990; Lovette & Holmes, 1995; Watts & Mitani, 2015). These methods are particularly susceptible to handling time, consumer motivation, and/or dependence on preference from resource availability and are subsequently difficult to validate against objective measures of abundance (Lovette & Holmes, 1995). The key advantage of the CCM is that rather than quantifying availability from occurrences of consumption (frequency dependent), the method depends on independent locations (spatially dependent), thereby allowing validation with traditional vegetation plot sampling.

Although we found a significant but minor periphery effect on agreement between methods in the presence/absence of species, the correlation of density estimates between methods was unaltered by the percentage of home range inclusion. The lack of a spatial effect on agreement between the methods is in some part likely to be a result of home range selection on the part of the consumer (e.g., second‐order selection sensu Johnson, 1980), that is, bonobos may have already selected their home range based on resource availability; hence, they show no sampling biases there within. In the absence of home range use biases, the CCM therefore reliably estimates resource availability across the entirety of a group's space use, although future studies should verify an absence of sampling biases on agreement between the CCM and traditional methods in their own study species.

Further, we found that consumption frequency of a species was correlated with the likelihood to miss species presences, that is, that infrequently consumed food species were also more likely to go unsampled in the dataset. Consequently, restricting estimation to only the top half of the consumed species (by frequency) appears to offer a compromise between maintenance of dietary relevance while maximizing fidelity with density estimates as assessed by objective plot measurements. This minimum translated to species consumed in approximately at least 60 locations over our 3‐year dataset. A general consequence of sampling frequency by a consumer is that estimates improve in precision as data accumulate over time. While species in our dataset were variable in “saturation level,” rates of new locations sampled by the bonobos slowed over the course of data collection and inter‐method correlation of species densities stabilized after fewer than 2 years of data collection (approximately 600 days).

Tree stands in the Kokolopori forest remained relatively stable, with the loss of monitored individuals averaging about 2.0% per year (range: 0.5%–3.7% between 2017 and 2021; E. Wessling & M. Surbeck, unpublished data). For locations like Kokolopori where tree stand is relatively stable from year to year, the CCM is likely to be able to provide estimates of species abundances within a reasonable margin of error. Intervals between vegetation plot surveys in animal studies are frequently longer than the CCM's 600‐day minimum, therefore the CCM may be better suited to adapt to environmental changes than plot sampling because users can restrict their data aggregation to a specified time window (as long as this window exceeds the minimum), thereby creating a dynamic measurement of availability that is continuously updated as new data accumulate. Such an approach would allow users to evade the duplication of surveying efforts required to capture tree stand changes using traditional plot sampling. In contrast, the CCM may not be suitable for research locations where tree stand is frequently disturbed (e.g., bushfires and anthropogenic disturbance), as the minimum necessary monitoring interval in CCM may be too long to account for abrupt or short‐term changes to the environment. In these environments, traditional plot sampling methods may be better suited as long as they are performed at commensurate intervals relative to individual resource turnover. As our results indicate that sampling rate affects the stability of estimates (e.g., frequency of consumption), we anticipate that this general minimum will be longer for species with slower sampling frequency, that is, for less frequently consumed, masting, or aseasonally consumed species. A myriad of other factors are likely to contribute to the speed at which data stabilize in methods like the CCM. We therefore recommend that researchers studying other systems evaluate the applicability of a method such as the CCM against resource stability and the traits of the consumer, relative to the suitability of more traditional methods (see Table 3).

**TABLE 3:** Advantages and disadvantages to the use of (a) traditional vegetation plot sampling and (b) the CCM for acquisition of information on food abundance to a consumer

Advantage	Disadvantage
(a) Vegetation plot sampling	
Provides an objective estimate of abundance of all potential tree species in a landscape	Effort may be wasted quantifying tree species that are irrelevant (i.e., ignored) to the consumer
Allows for the quantification of landscape‐level characteristics non‐specific to the consumer such as overall species richness, total tree density, and total basal area	Choice of method used may inhibit ability for cross‐site comparison when different methods are used may introduce biases or errors toward certain characteristics of measured species
Methodology can be adjusted and tailored to different end goals and to accommodate various characteristics in the environment or survey targets (trees/lianas)	Survey effort may need to be intensive depending on desired outcomes (e.g., if species of interest are rare, landscape is large, or detailed sub‐landscape comparison is needed)
Generally comparable across landscapes and objective (i.e., non‐specific) to the landscape rather than particular consumers (e.g., study species) or social units	Is a static measurement of a single snapshot in time —survey area must be resurveyed if changes in the area occur
No “burn‐in” time required: data are immediately useable once minimum sampling is met	Can only approximate the distribution of individuals at a scale fixed to the methodology—requires a priori assumptions of relevant scales of distribution to a consumer
Does not necessitate direct observation of the consumers	Can measure only abundance but cannot provide information on distribution of potential feeding locations or actual availability of resources to a consumer
Is independent of consumer movement, therefore sampling can target areas of interest	
Able to measure dispersion using finite and spatially explicit samples	
(b) Consumer‐Centric Method (CCM)	
For frequently consumed species, could it theoretically be capable of providing a census of all relevant individuals of a given species once data are fully saturated	Data are not generalizable beyond the sampled individuals or social group
Provides temporally dynamic monitoring of distribution of visited (i.e., relevant) feeding locations; can reflect changes within the area of interest over time	For now, only appears suitable for quantification of densities and some species' distributions; traditional methods may still be required if other metrics are desired
Provides dynamic monitoring of availability of both abundance as well as consumer behavior changes or changes in selection	Information gained is limited only to consumed species
Data are able to reflect the true availability of resources rather than abundances (which are blind to patterns of use and temporally varying variability)	Quality of information may be biased toward frequently consumed species
Because they are targeted by the consumer, the CCM may allow for better capture rates of species otherwise rare in the landscape	Requires a “burn in” period before reliable and stable estimates can be provided and data are of sufficient depth
Tailored directly to social unit (e.g., individual and community) and reflects selection biases inherent to each social unit	Requires direct behavioral observation of the consumer
Data are collected directly at the scale most relevant to the consumer and are therefore not aggregated to impose ad hoc scales of summarization	Currently requires cross‐validation with traditional plot sampling before the method is demonstrated to be robust across contexts
Is easily integrated into existing behavioral observation data collection and does not require supplementary data collection	Is not a valid method when an assessment of resource preference or selection is relevant to the study question
With data collection teams sufficiently trained in botanical identification of all food items, does not require additional research effort from botanists	Is the joint outcome of resource selection and true abundance, therefore cannot disentangle changes in either input from the other

Generally, species distribution (i.e., spatially explicit relative abundance) correlated weakly between the methods across species regardless of the scale of comparison (i.e., cell size). A greater proportion of species reached significant agreement between methods in smaller rather than larger cell sizes, potentially as a function of proximity between bonobo foraging behavior and sampled plots (i.e., the larger the cell size used, the greater the potential distance between bonobo feeding locations and comparatively small plot areas). Nevertheless, our finding that correlations of distribution within species were significant across a greater proportion of food species than expected by chance (i.e., 5%) and that the rates at which bonobos missed the presence of a species in a cell are likewise better than common rates of species misses between multiple observers sampling the same plot (Milberg et al., 2008) provides hope that reliable estimates of sub‐landscape abundances and presence distribution may improve with greater sampling depth.

While detectability is rarely 100% in either method (Morrison, 2016), the miss‐rates by a consumer in the CCM may rather carry additional information about the nature of resource selection (and the individuals that are subsequently ignored). This is especially likely to be the case in consumers who have the capacity to keep track of spatiotemporal patterns of resource availability. Bonobos likely have a concept of where and when resources become available, and therefore are also capable of targeting resources that are rare (Janmaat et al., 2013; Normand et al., 2009). Consequently, the CCM mimics ad hoc sampling (Foster et al., 1998; Gordon & Newton, 2006; Hopkins, 2007), and our results indicate that the CCM more closely matches Plot Density estimates at capturing rare species relative to more abundant species.

Nonetheless, in the absence of full censusing, we cannot differentiate which sampling method produced a more precise representation of food species availability, dispersion, and distribution patterns. Ideally, methodological sampling biases could be identified by simulating both sampling schemes from a simulated “forest”. However, as we rarely understand the complexity of consumer movement and resource selection patterns (Buskirk & Millspaugh, 2006), subsequent conclusions drawn from simulated sampling behavior would be just as arbitrary as the decisions made to simulate them (Johnson, 1980).

### 4.2 Measuring different phenomena

We argue that the CCM, with adequate evaluation, may be a more appropriate tool for some applications in behavioral ecology than traditional inventory methods such as plot sampling. Traditional plot sampling quantifies the total amount of potential resources including inaccessible, unattractive, or otherwise unpalatable resources to a consumer. Only a subset of these resources comprises true resource availability, that is, resources with potential to be selected (Alldredge et al., 1998; Buskirk & Millspaugh, 2006; Johnson, 1980), and although correlated, each represents inherently separate phenomena (Hutto, 1990). Because we rarely understand the processes of food selection by which consumers filter objective resource abundance into availability, the CCM offers the advantage of using the consumer as a means to avoid arbitrary decisions as to how to best sample the landscape (Johnson, 1980). We detail examples of this selectivity and the resulting advantages of the CCM below.

First, we observed significant differences between average sizes of trees/lianas visited by bonobos relative to what was available in the landscape of consumed species (as measured in vegetation plots). Reducing our plot dataset to a single selection criterion (tree/liana sizes selected by the consumer) increased the correlations between CCM and vegetation plot measures by an average of 4%–7% as a simple demonstration of the inadequacies of consumer‐objective plots in mirroring consumer behavior. Second, that bonobos missed or ignored certain food resources in cells identified to contain them underlines further how researchers are likely unaware of relevant selection criteria that impact the measurement of true resource availability. Because apes possess mental maps of their environments and are known to adjust travel to target preferred food sources (Janmaat et al., 2013; Lucchesi, Cheng, Wessling, et al., 2021), they are unlikely to consistently miss available food resources within their home range over extended periods of time. In a future study, this may be confirmed by evaluating if particular cells within the home ranges consistently disagreed between methods across food species. Third, we found that CCM estimates of density and distribution differed between bonobo social groups, even with largely overlapping home ranges. This conforms to previous findings of group‐specific feeding selection criteria in bonobos (Samuni et al., 2020), independent of local abundance. If resource availability for a consumer in a given landscape is dependent on group identity, then only methods like the CCM incorporating these criteria allow comparable estimates for comparative studies across social groups.

Altogether, by accounting for consumer selection, the accumulation of data on food patch location is inherently less subjective than datasets dependent on arbitrary decisions by the investigator (Johnson, 1980) because it does not involve decisions by the investigator about selection criteria. However, such a method precludes its ability to be used for assessing the components involved in selection by a consumer and preference if conducted in the absence of objective abundance sampling (e.g., plots). Conversely, when conducted in parallel to plot sampling, the CCM can provide insight into which resources are regularly ignored, and consequently, the components leading to biases in consumption (i.e., availability relative to abundance). It should be noted, that biases in resource measurement in consumer‐objective sampling also occur via multiple channels including selection of sampling method, metric, and effort, as well as through unavoidable systematic or random measurement errors (Baraloto et al., 2013; Milberg et al., 2008; Morrison, 2016; Ståhl et al., 2017; Wessling et al., 2020). The CCM, however, accounts for several of these issues because consumers are knowledgeable and motivated surveyors who actively target resources, with apparently negligible impact of scale variation (e.g., cell size) or abundance on fidelity of CCM estimates to plot‐derived estimates. Therefore, estimates derived from the CCM could theoretically provide accurate measures of availability once data have reached a sufficient depth.

Difficulties with GPS signal in Kokolopori forced us to coarsen data precision to 50 × 50 m quadrats, and consequently, a loss of fine‐tuned information on actual abundance of resources visited. Subsequently, our results mimic the same difficulties identified by Jiménez‐Valverde et al. (2021) in the fidelity of presence‐only‐derived abundance estimates to true abundance. Future studies that are able to track visits to specific resource patches will likely allow for more honest sampling of the abundance of resources visited and may permit CCM datasets to avoid suffering the same shortcoming. Nonetheless, the CCM was able to correlate with density estimates provided by traditional plot sampling at a rate of up to 69%, suggesting moderate but imperfect comparison between the two methods. That the two methods correlate only moderately well may suggest that they likely measure similar but different resource groupings (potential vs. used). Although in this study we cannot confirm that the CCM measures true availability with greater precision than plot sampling, it anyways remains to be validated that plot sampling can provide estimates reflective of measures of true availability either.

Our spatially explicit CCM further allows for data accumulation and consequential improvement in the accuracy of estimates over time until otherwise removed due to irrelevance (e.g., patch loss). Nevertheless, if rapid density assessment is preferable for a project, traditional ecological sampling may remain a preferred method due to a 600 person‐day burn‐in time required (this study) by the CCM before estimates become reliably stable per social group relative to 150 person‐days of plots for both groups. However, these 150 person‐days are supplementary to observational data, insomuch as person‐days necessary to collect both sets of data must be considered additive to observational data collection. Yet, if databases of feeding locations are already available, adapting these data to CCM estimation of resource density or distribution saves researchers from needing to collect additional data to quantify resource abundance.

While this method is best applied to estimate the availability of discrete, immobile, and spatially explicit resources, these advantages transcend application beyond bonobos and allow researchers to evaluate the strengths of the method for their investigations across all potential consumers who meet these criteria (further discussed in Table 3). Functionally, assumptions of the CCM are similar to studies investigating resource preference, a method that also combines objective habitat measures with subjective animal‐centric data (Manly et al., 2007), and requires space re‐use for data to aggregate. Researchers must verify whether their existing or potential datasets to be used for CCM sampling are of sufficient sampling depth and absent of biases (e.g., sampling biases or characteristics of food items) for their consumers before the CCM can be applied as a means of replacing objective resource measurement with the CCM for resource availability.

Here, we offer a context‐specific evaluation in two social groups of a tropical frugivore of the CCM, a data collection method allowing researchers to quantify resource availability to a consumer. It serves as a potential new tool for animal behavior studies, and our results offer a roadmap for when and how such a methodology may be useful in other contexts and models. Many factors are likely to affect the fidelity of the CCM to true resource availability in a landscape, and the characteristics of both the consumer and the resource will impact the relative advantages of applying the method over traditional plot sampling (Table 3). For now, in the absence of robust validation of the method across food type, landscapes, and study species, researchers interested in applying the CCM to their research contexts should still perform traditional vegetative sampling and validate their results between these two datasets before fully committing to the use of the CCM only. Once this initial hurdle is surpassed, the CCM allows continuous and comprehensive sampling of relevant resources within a consumer's environment that barring significant changes to the landscape or consumer preference will provide a dynamic and updateable estimate with little additional effort. When applied correctly, the CCM will enable many behavioral ecologists to quantify aspects of food availability by using data already existing in their research repertoires. Furthermore, resource metrics derived from the CCM may be more suitable to its application as well as allow for more precise comparison in ways that make these data comparable across social groups, subsequently promising new insights into the interplay between an animal and its environment. Further validation will illuminate the applicability and appropriateness of new methods like the CCM in replacing pervasive but imperfect methodologies like plot sampling as wild animal research seeks more accurate and efficient methodologies in capturing animal behavior and the forces that affect it.

## AUTHOR CONTRIBUTIONS

**Liran Samuni:** Conceptualization (supporting); formal analysis (supporting); investigation (supporting); methodology (supporting); writing – review and editing (supporting). **Roger Mundry:** Formal analysis (supporting); investigation (supporting); methodology (supporting); writing – review and editing (supporting). **Miguel Adan Pascual:** Data curation (supporting); investigation (supporting); writing – review and editing (supporting). **Stefano Lucchesi:** Data curation (supporting); investigation (supporting); writing – review and editing (supporting). **Bienfait Kambale:** Validation (supporting); writing – review and editing (supporting). **Martin Surbeck:** Conceptualization (supporting); funding acquisition (lead); investigation (supporting); methodology (supporting); resources (equal); writing – review and editing (supporting). **Erin G. Wessling:** Conceptualization (lead); data curation (lead); formal analysis (lead); investigation (lead); methodology (lead); writing – original draft (lead); writing – review and editing (lead).

## CONFLICT OF INTEREST

The authors have no conflict of interest to declare.

## Supporting information


Appendix S1.

## Data Availability

All necessary data to reproduce this study will be submitted to the Dryad Digital Repository.
